# MicroRNA-188-5p inhibits hepatocellular carcinoma proliferation and migration by targeting forkhead box N2

**DOI:** 10.1186/s12885-023-10901-7

**Published:** 2023-06-05

**Authors:** Yan-hui Wu, Bin Yu, Jiang-min Zhou, Xue-han Shen, Wei-xun Chen, Xi Ai, Chao Leng, Bin-yong Liang, Ya-jie Shao

**Affiliations:** 1 Hepatic Surgery Center, Tongji Hospital, Tongji Medical College, Huazhong University of Science and Technology, 1095 Jiefang Avenue, Wuhan, Hubei 430030 China; 2 Department of General Surgery, the First Affiliated Hospital of Nanchang University, Nanchang, 330006 China; 3 Department of Anesthesiology, Tongji Hospital, Tongji Medical College, Huazhong University of Science and Technology, 1095 Jiefang Avenue, Wuhan, Hubei 430030 China

**Keywords:** HCC, Metastatic, miR-188, FOXN2, Proliferation

## Abstract

**Background:**

This study aimed to identify the biological functions, expression modes, and possible mechanisms underlying the relationship between metastatic human hepatocellular carcinoma (HCC) and MicroRNA-188-5p (miR-188) dysregulation using cell lines.

**Methods:**

A decrease in miR-188 was detected in low and high metastatic HCC cells compared to that in normal hepatic cells and non-invasive cell lines. Gain- and loss-of-function experiments were performed in vitro to investigate the role of miR-188 in cancer cell (Hep3B, HepG2, HLF, and LM3) proliferation and migration.

**Results:**

miR-188 mimic transfection inhibited the proliferation of metastatic HLF and LM3 cells but not non-invasive HepG2 and Hep3B cells; nonetheless, miR-188 suppression promoted the growth of HLF and LM3 cells. miR-188 upregulation inhibited the migratory rate and invasive capacity of HLF and LM3, rather than HepG2 and Hep3B cells, whereas transfection of a miR-188 inhibitor in HLF and LM3 cells had the opposite effects. Dual-luciferase reporter assays and bioinformatics prediction confirmed that miR-188 could directly target forkhead box N2 (FOXN2) in HLF and LM3 cells. Transfection of miR-188 mimics reduced FOXN2 levels, whereas miR-188 inhibition resulted in the opposite result, in HLF and LM3 cells. Overexpression of FOXN2 in HLF and LM3 cells abrogated miR-188 mimic-induced downregulation of proliferation, migration, and invasion. In addition, we found that miR-188 upregulation impaired tumor growth in vivo.

**Conclusions:**

In summary, this study showed thatmiR-188 inhibits the proliferation and migration of metastatic HCC cells by targeting FOXN2.

**Supplementary Information:**

The online version contains supplementary material available at 10.1186/s12885-023-10901-7.

## Background

Hepatocellular carcinoma (HCC) is a primary malignancy of the liver that represents 80% of all primary liver cancers worldwide [1]. Most patients are only firmly diagnosed with HCC at the terminal stage, and because of this, the optimum time for radical treatment is often missed, particularly in less-developed countries. Overall HCC prognosis is unfavorable because of early recurrence or metastasis even with excellent treatment [2, 3]. Thus, it is crucial to explore and understand the relevant molecular mechanisms of HCC development to discover novel treatment targets. 

miRNAs function as oncogenes or tumor inhibitors of HCC, which facilitates mRNA degradation of their target genes as a way of after-transcription modulation to suppress the translation of these target genes [4]. miRNAs can serve as either tumor inhibitors or oncogenes, depending on the corresponding target mRNAs [5]. Many studies have shown that miRNAs can specifically bind to target genes to modulate the biological processes and pathologies of diverse malignant tumor cells [6, 7]. Several studies have shown that miR‐188 is a new tumor‐inhibitor in breast cancer, prostate cancer, oral squamous cell cancer, colorectal cancer papillary thyroid cancer, colon cancer, bladder cancer, and glioma [8]. In addition, miR‐188 could also serve as a tumor inhibitor in HCC by targeting FGF5 [9], lncRNA CASC11 [10], and lncRNA PAPAS [11]. Although dysregulation of miR‐188 has been reported to be associated with HCC, the biological activity of this gene in HCC cell lines with diverse metastatic properties is still unknown. This study explored the tumor-suppressor role of miR‐188 in HCC cells with diverse metastatic potentials and predicted its target gene.

## Methods

### Patients and follow-up

In this study, 94 HCC patients (50 males and 44 females, 37–71 years, 50.0 ± 6.3 years) were included and were admitted to Tongji Hospital, Tongji Medical College, Huazhong University of Science and Technology (2016. 6 ~ 2019. 5) Inclusion criteria were as follows: (1) first-time diagnosis; (2) no prior treatment; (3) agreement to be followed up. The exclusion criteria were as follows: (1) other clinical diseases, (2) prior treatment, and (3) a history of malignant cancers. The results indicated that 31 were HBV-positive, 29 were HCV-positive, and the others were negative for both. The present study excluded patients who exhibited positive results in both groups. Prior to treatment, a liver biopsy was conducted, and HCC and adjacent healthy tissues were harvested and confirmed by no fewer than three experienced pathologists. The follow-up was continued for 4 years, and survival data were obtained via telephone or outpatient visits. Patients who died for other reasons or were lost to follow-up were excluded. All subjects signed an informed consent form, and the study was approved by the Ethics Committee of Tongji Hospital, Tongji Medical College, Huazhong University of Science and Technology.

### Cell lines

Human HCC cell lines, including non-invasive Hep3B and HepG2, low-metastatic Huh7, Bel7402, HLE, HLF, and SMMC7721, metastatic HCC97H and HCC-LM3, and human hepatocyte QSG7701 and HL7702 cells, were used in the cell experiments. Cell lines were provided by the ATCC and cultivated in RPMI 1640 medium with 10% FBS in an environment of 5% CO_2_ at 37 °C.

### qPCR

To determine miR-188 expression, mRNAs were isolated, reverse transcription was conducted using a microRNA cDNA Synthesis Kit (Qiagen), and the PCR reaction system was obtained using the SYBR Green PCR Kit (Qiagen). To determine *FOXN2* expression, total RNA was isolated with TRIzol, cDNA was obtained using SuperScript III Reverse Transcriptase, and all PCR reaction systems were prepared using the One‐Step RT‐qPCR Kit (NEB). MiR-188 and *FOXN2* expression levels were normalized to those of U6 and *GAPDH*, respectively. All results were normalized using the 2 − ΔΔCT method.

### Cell transfection

miR-188 mimic/inhibitor and the corresponding negative control (NC mimic/inhibitor) (GenePharma, Shanghai, China) at a concentration of 40 nM were transfected into 4–6 × 10^5^ HCC cells using Lipofectamine 3000 (Invitrogen, Carlsbad, CA, USA).

### Cell proliferation and colony formation assay (CFA)

The cell proliferation capacity was evaluated using the CCK-8 assay with the relevant guidance. After inoculation into 96-well plates supplemented with CCK-8 (10 μl), cells were subsequently incubated at 37 °C for an additional 2 h. OD_450 nm_ (optical density) was determined using an Infinite M200 (Tecan, Switzerland). For the CFA experiment, cells were cultivated in 6-well plates for 7 d, fixed in formaldehyde (4%) for 20 min, and stained with crystal violet (1.0%).

### Cell migration assays

An in vitro migration assay was conducted using transwell plates with 8 µm pores. Cells (1 × 10^4^) in RPMI 1640 were transferred to the upper chamber. RPMI 1640 medium containing 5% FBS was added to the lower chamber as a chemoattractant. After incubation for 2 d, the cells were cleared with cotton swabs from the upper surface, and the migratory cells were fixed in MeOH and stained with crystal violet (0.5%) at RT for 0.5 h. Images were taken, and cell counting was conducted with a × 200 light photomicroscope.

### Dual-luciferase reporter assay (DLRA)

WT and Mut 3ʹ-UTRs of *FOXN2* were subjected to DLRA to identify whether miR-188 targets *FOXN2*. Based on the firefly luciferase sequences, fluorescence was calibrated using *Renilla* luciferase as a reference. After transfection with the miR-188 mimic/NC mimic and fluorescence vectors, the cells were incubated for 72 h.

### TargetScan prediction

The TargetScan prediction algorithm was used to identify the targets of miR-188. Predictions are listed as targeting efficacy predictions on the website http://www.targetscan.org [12]. Predictions were ranked based on the possibility of conserved targeting [13].

### Preparation of doxycycline-inducible constructs and stable cell selection

Doxycycline-inducible constructs were prepared according to relevant guidelines. cDNA oligos for *FOXN2* ORF were prepared to obtain a Tet-on/FOXN2 overexpress plasmid and cloned into the pSingle-tTS-FoxN2 vector at XhoI/HindIII sites (Clontech), and sequencing was conducted for recombinant clones. Cells were exposed to pSingle-tTS-Luciferase (Tet-on/Luc, vector control) or pSingle-tTS- *FOXN2* (Tet-on/*FOXN2*) and selected with 0.5 mg/ml G418 for 2 weeks. G418-resistant cells (HLF and LM3) were isolated and grown. Doxycycline (1 μg/ml, Sigma) was added to the cell culture medium with Tet system-approved FBS (10%, Clontech) to overexpressed FOXN2 expression.

### Data analysis

The results are displayed as the mean ± SD. Data were analyzed using SPSS 17.0. One-way ANOVA followed by Tukey’s post-hoc test and Student’s t-tests were used to evaluate the differences among multiple groups and the distinction between two groups, respectively. Survival curves were plotted using the Kaplan–Meier method, followed by the log-rank test based on overall survival and FOXN2 relative expression. Statistical significance was set at *P* < 0.05. All experiments were conducted in triplicate.

## Results

### miR-188 is decreased in HCC samples and metastatic HCC cell lines

**Fig. 1:**
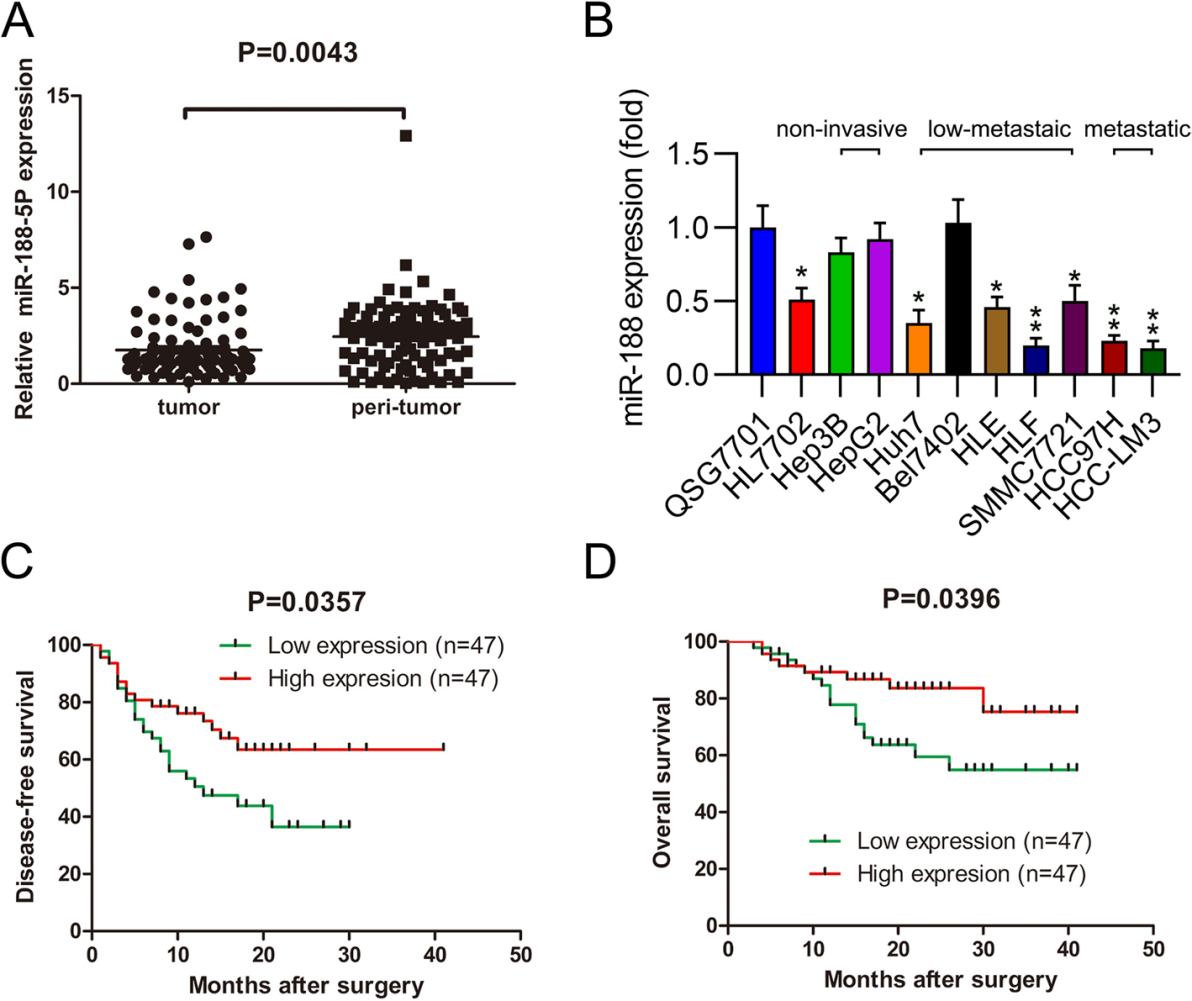
Expression of miR-188 in hepatocellular carcinoma (HCC) cell lines and noncancerous cells. **A** Relative expression of miR-188-5p in HCC tumor and peri-tumor. **B** qPCR was performed to detect miR-188 levels in noncancerous hepatic cells (QSG7701 and HL7702) and HCC cell lines (non-invasive Hep3B and HepG2, low-metastatic Huh7, Bel7402, HLE, HLF, SMMC7721, and metastatic HCC97H and HCC-LM3 cells). **C** Kaplan–Meier analysis of disease-free survival of 94 HCC patients according to miR-188-5p expression levels in HCC tissues. **D** Kaplan–Meier analysis of overall survival of 94 HCC patients according to miR-188-5p expression levels in HCC tissues.* *P* < 0.05, ** *P* < 0.01

miR-188 expression in HCC and adjacent healthy samples was detected using qPCR. Relative to levels in the latter, miR-188 was dramatically downregulated in 94 HCC samples (Fig. 1A). Subsequently, miR-188 expression in human HCC cell lines, such as non-invasive Hep3B and HepG2, low-metastatic Huh7, Bel7402, HLE, HLF, and SMMC7721, metastatic HCC97H and HCC-LM3, and human hepatocyte QSG7701 and HL7702 cells was compared. These results showed that miR-188 levels in the other HCC cell lines were significantly reduced when compared with QSG7701 cells. However, in non-invasive HepG2 and Hep3B cells, miR-188 showed no statistically significant difference (Fig. 1B).

### Low miR-188 levels in HCC samples are tightly related to low survival

Patients with HCC (94) were classified into high and low miR-188 expression groups based on miR-188 expression as with Youden’s index, and each group included 47 patients. The K-M method and log-rank test were used to plot and compare the survival curves. The results indicated that the low miR-188 level group exhibited a dramatically lower DFS rate (Fig. 1C) and overall survival rate (Fig. 1D).

### miR-188 dysregulation in HCC cells (HepG2, Hep3B, HLF, and LM3)

**Fig. 2:**
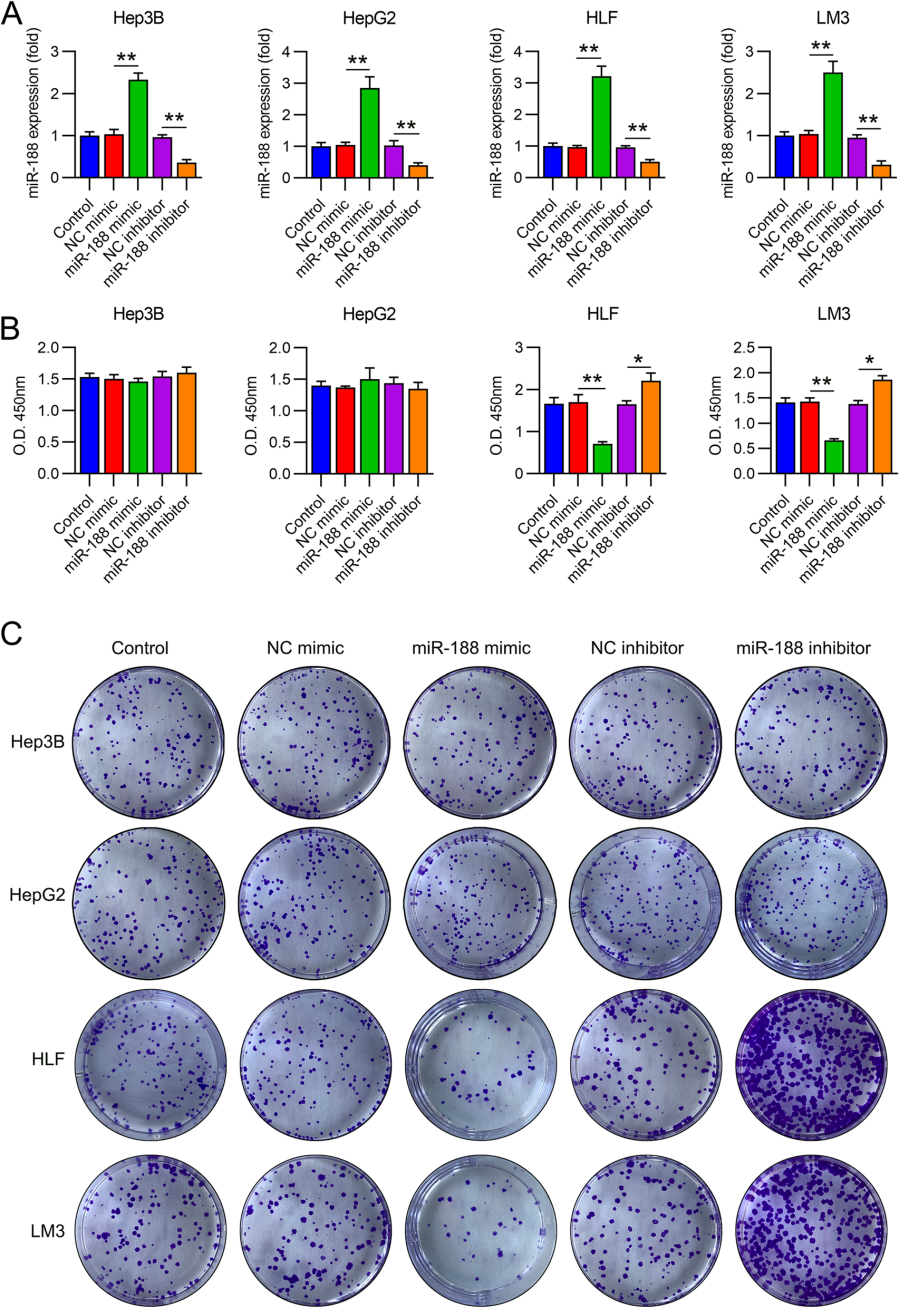
Role of miR-188 on growth and colony formation of hepatocellular carcinoma (HCC) cell lines. HepG2, Hep3B, LM3, and HLF cells were transfected with the miR-188 mimic/inhibitor or NC mimic/inhibitor for 72 h. **A** At 72 h post transfection, qPCR was carried out to detect miR-188 levels in HLF, LM3, Hep3B, and HepG2cells. **B** HepG2, Hep3B, LM3, and HLF cells were transfected with miR-188 mimic/inhibitor or NC mimic/inhibitor for 72 h. CCK-8 assays were used to determine cell viability at 72 h post-transfection. **C** Colony formation assays were used to determine the growth rates of these four HCC cell lines. * *P* < 0.05, ** *P* < 0.01

Next, we attempted to evaluate the effect of miR-188 on HCC cells by transfecting miR-188 mimic/inhibitor into non-invasive cell lines (HepG2 and Hep3B) and metastatic cell lines (HLF and LM3) for 48 or 72 h. RT-qPCR indicated that miR-188 levels were significantly upregulated in these four cell lines (Fig. 2A) at 72 h after miR-188 mimic transfection, while miR-188 inhibitor transfection did attenuate miR-188 levels in cells (Fig. 2A).

### miR-188 inhibits cell proliferation of HLF and LM3 cells but not HepG2 and Hep3B cells

We then explored the effect of miR-188 on the viability and proliferation of HCC cell lines. The CCK-8 assay indicated that HLF and LM3 cell, but not HepG2 and Hep3B cell, proliferation rates were significantly reduced at 3 d following miR-188 mimic transfection, relative to those in the control groups (Fig. 2B). Further, miR-188 inhibitor transfection enhanced the growth of HLF and LM3 cells (Fig. 2B). Colony formation assays were then performed to confirm that miR-188 upregulation could dramatically decrease the number of colonies formed by HLF and LM3 cells; nevertheless, miR-188 downregulation promoted colony formation by HLF and LM3 cells. Expectedly, in HepG2 and Hep3B cells, miR-188 dysregulation did not have an effect (Fig. 2C), suggesting that miR-188 inhibits the proliferation of HLF and LM3 cells.

### MiR-188 suppresses cell migration and invasion of HLF and LM3 cells but not HepG2 and Hep3B cells

**Fig. 3:**
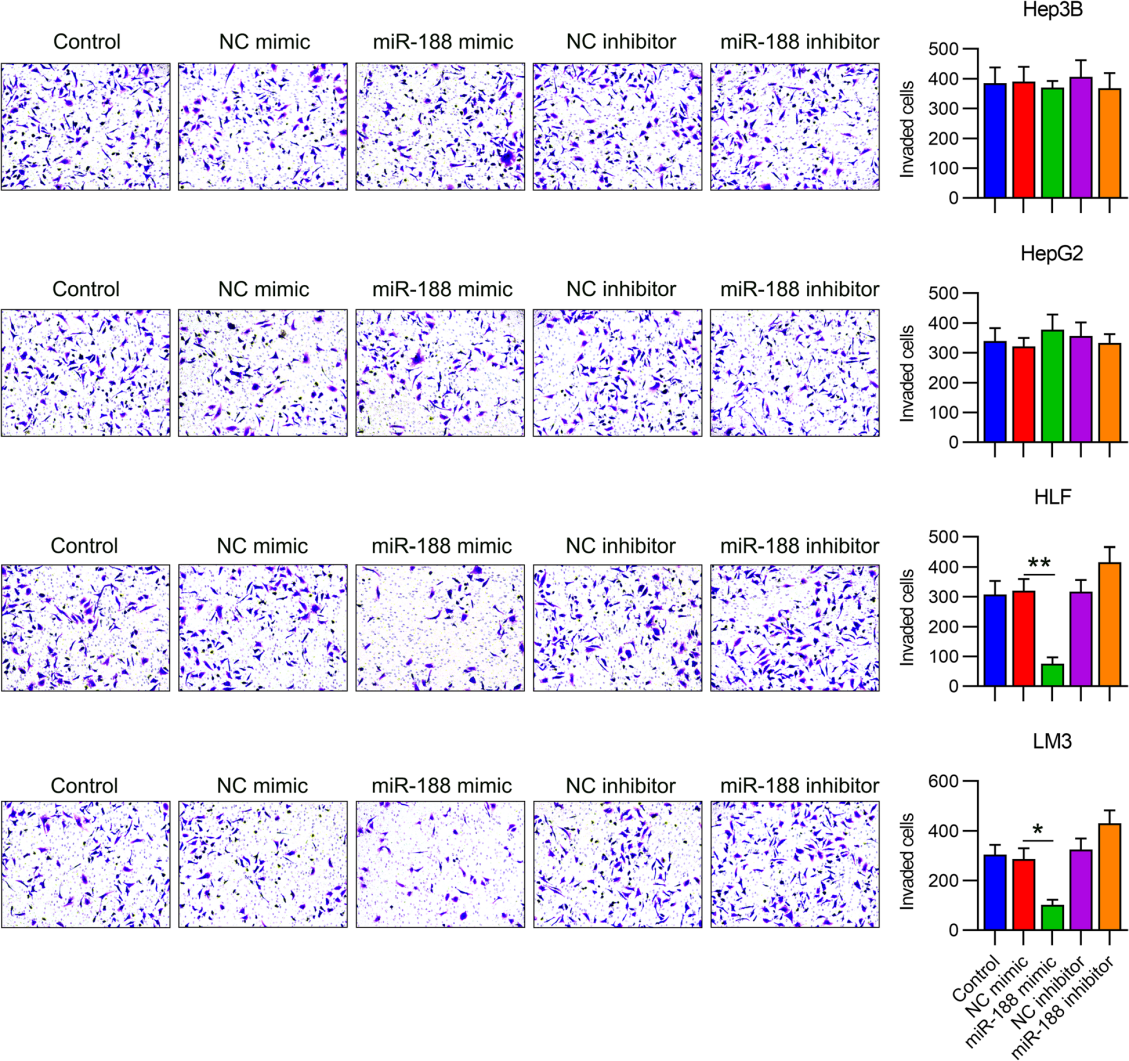
Invasion of hepatocellular carcinoma (HCC) cell lines with dysregulated miR-188. HepG2, Hep3B, LM3, and HLF cells were transfected with miR-188 mimic/inhibitor or NC mimic/inhibitor for 72 h. Transwell invasive assays of HCC cells with miR-188 overexpression or inhibition. * *P* < 0.05, ** *P* < 0.01

**Fig. 4:**
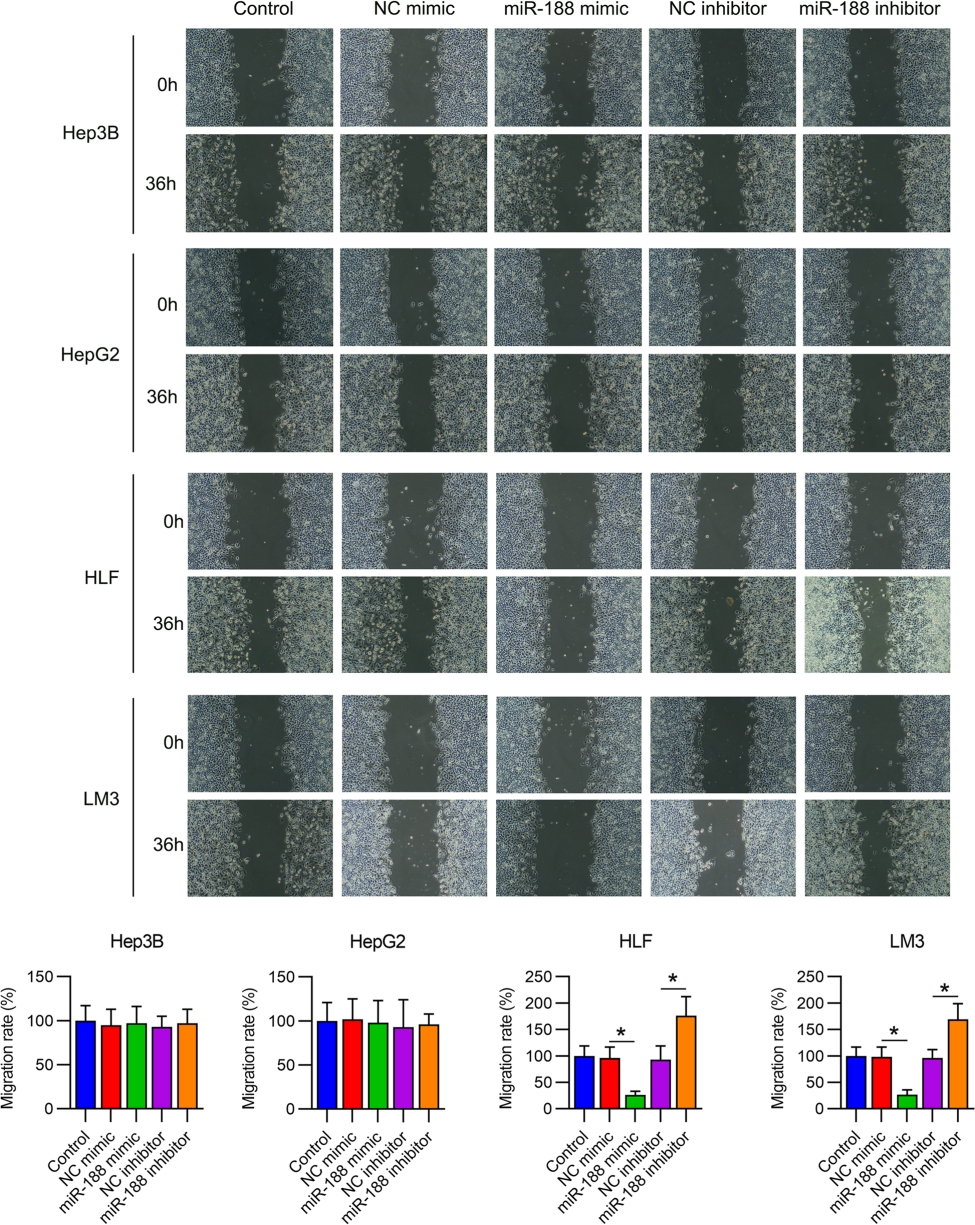
Migration of hepatocellular carcinoma (HCC) cell lines with dysregulated miR-188. HepG2, Hep3B, LM3, and HLF cells were transfected with miR-188 mimic/inhibitor or NC mimic/inhibitor for 72 h. The migratory percentage of cells was determined by wound healing assay. * *P* < 0.05

To determine the role of miR-188 in migration and invasion, a transwell migration and wound healing assay was conducted with HLF, LM3, HepG2, and Hep3B cells. The results showed that miR-188 upregulation dramatically reduced the migration and invasive rates of HLF and LM3 cells, rather than Hep3B and HepG2 cells (Fig. 3). We also found that miR-188 downregulation promoted cell migration of HLF and LM3 cells (Fig. 4). Therefore, these results showed that, consistent with the proliferation trend, miR-188 inhibited migration and invasion of metastatic HCC cell lines.

### MiR-188 targets the 3′-UTR of the *FOXN2* gene

**Fig. 5:**
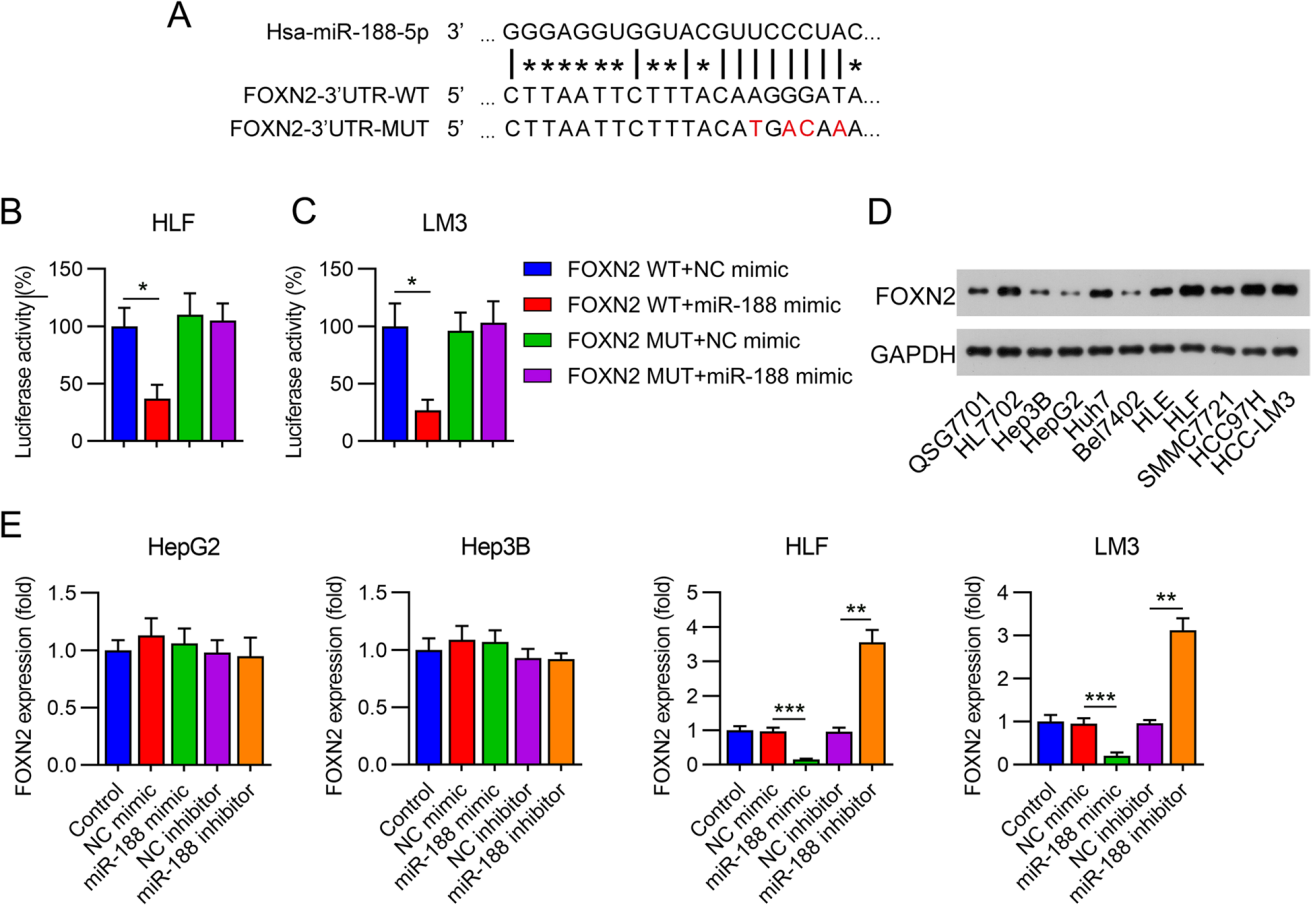
FOXN2 is a direct downstream target for miR-188 in hepatocellular carcinoma (HCC) cells. **A** The sequences of predicted miR-188-binding sites within the 3ʹ-UTR of *FOXN2*; the wild-type (WT) or mutant (Mut) binding sites are shown. **B**, **C** Relative luciferase activity was analyzed after the reporter plasmids or mock reporter plasmid with miR-188 mimics were co-transfected into LM3 and HLF cells. **D** WB was performed to detect FOXN2 levels in noncancerous hepatic cells (QSG7701 and HL7702) and HCC cell lines (non-invasive Hep3B and HepG2, low-metastatic Huh7, Bel7402, HLE, HLF, SMMC7721, and metastatic HCC97H and HCC-LM3 cells). **E** HepG2, Hep3B, HLF, and LM3 cells were transfected with miR-188 mimic/inhibitor or NC mimic/inhibitor for 72 h. qPCR was performed to assess the mRNA level of *FOXN2* in. * *P* < 0.05, ** *P* < 0.01, *** *P* < 0.001

Bioinformatic prediction was subsequently conducted to identify the putative miR-188 target. To investigate the specific correlation between miR-188 and genes, Targetscan (www.targetscan.org/mamm_31/) and miRanda (www.microrna.org/microrna/home.do) were performed. The FOXN2 was selected to be the predicted targeting of miR-188. The fragments of the 3′-UTR of FOXN2 contains miR-188 binding sites. The results suggested that the FOXN2 3′-UTR has miR-188-binding sites (Fig. 5A). DLRA results further showed that *FOXN2* is an essential target gene of miR-188, and miR-188 mimic transfection suppressed luciferase activity by targeting the *FOXN2* WT 3′-UTR in HLF and LM3 cells (Fig. 5B, C). Then, FOXN2 expression in human HCC cell lines, including non-invasive Hep3B and HepG2, low-metastatic Huh7, Bel7402, HLE, HLF, and SMMC7721, metastatic HCC97H and HCC-LM3, and human hepatocyte QSG7701 and HL7702 cells was examined by WB. These results showed that FOXN2 levels in the HLF, HCC97H, and LM3 cell lines were significantly increased when compared with other cells (Fig. 5D, Supplementary Fig. 1). Meanwhile, the expression of FOXN2 in these HCC cell lines is negative correlated to the expression of miR-188 (Fig. 1B). To further confirm the correlation between miR-188 and *FOXN2* expression, qPCR was performed to examine *FOXN2* mRNA expression in cells transfected with mimics/inhibitors. RT-qPCR revealed that the miR-188 mimic downregulated *FOXN2* levels in cells. Meanwhile, miR-188 downregulation led to increased *FOXN2* mRNA expression in HLF and LM3, other than in Hep3B and HepG2 cells (Fig. 5E), suggesting that *FOXN2* expression is inversely correlated with miR-188 expression, and explained why miR-188 only exerted its function in HLF and LM3 cells.

### Effect of FOXN2 knockdown on miR-188-mediated proliferation and migration of LM3 and HLF cells

**Fig. 6:**
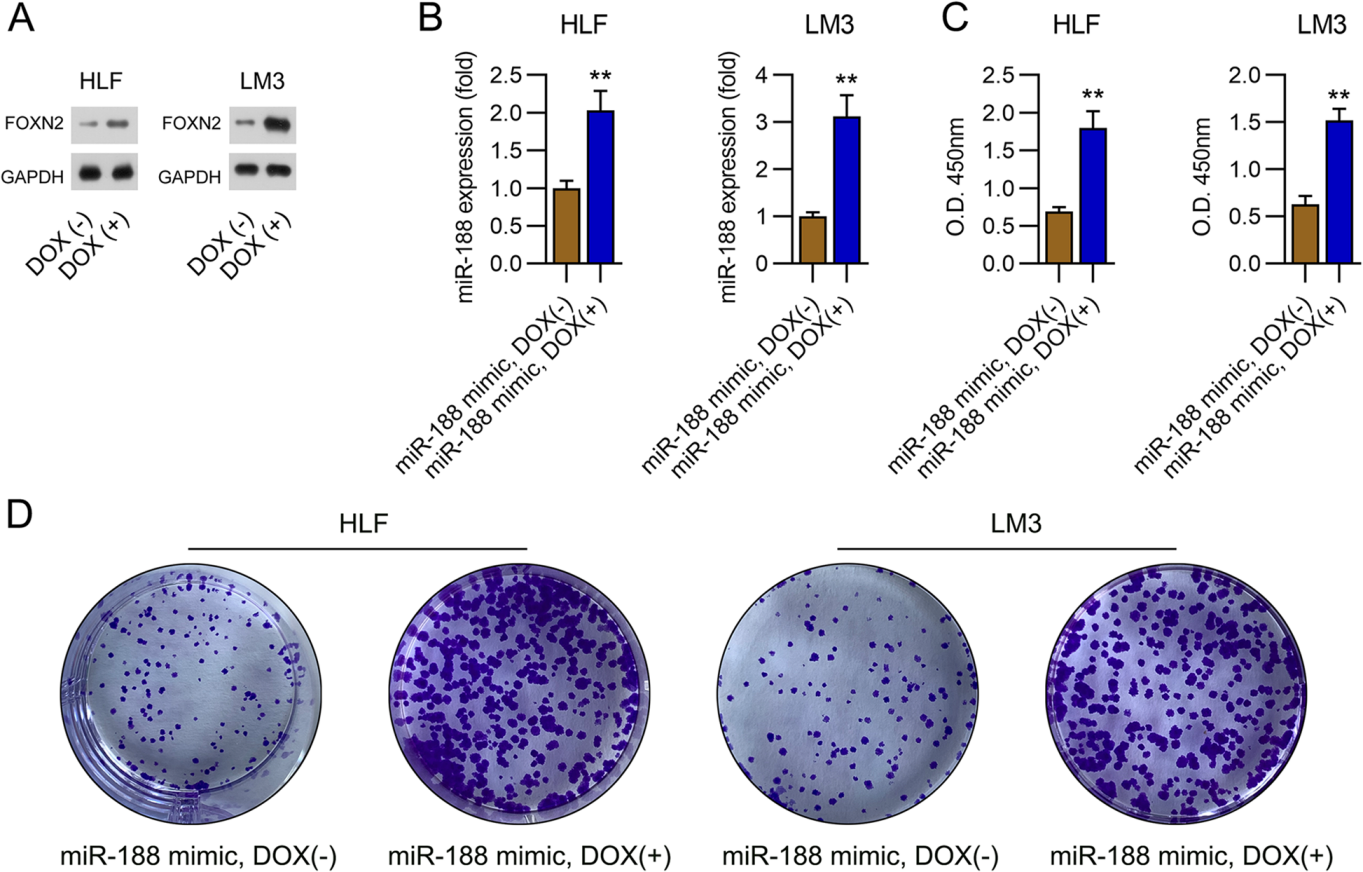
Stable upregulation of FOXN2 on growth of HLF and LM3 cells. **A** HLF and LM3 cells stably expressing FOXN2 were treated with doxycycline (Dox, 50 ng/ml). After 166 h, the cells were harvested and subjected to RT-qPCR. The expression level of *FOXN2* mRNA was determined by PCR. **B** HLF and LM3 cells with miR-188 mimic transfection was treated with doxycycline (Dox, 50 ng/ml). After 166 h, the cells were harvested and subjected to qPCR. The expression level of *FOXN2* mRNA was determined by qPCR. **C** The CCK-8 assay was used to determine cell viability at 72 h post-transfection. **D** The cell growth rate was determined by colony formation assay. ** *P* < 0.01

To further confirm the influence of FOXN2 on the malignant properties of HCC cells with miR-188 upregulation, a Tet-on inductive system was used to stably overexpressed FOXN2 in LM3 and HLF cells. After doxycycline treatment, FOXN2 expression in these two cell lines was overexpressed in cells with or without miR-188 upregulation (Fig. 6A, B, Supplementary Fig. 1). CCK-8 assays were subsequently conducted to detect cell proliferation at 72 h following doxycycline treatment. The results showed that the proliferation rates were significantly upregulated as a result of FOXN2 overexpression, as compared with those in miR-188-uprgulated LM3 and HLF cells (Fig. 6C, D). These data indicated an oncogenic role of FOXN2 in LM3 and HLF cell proliferation.

**Fig. 7:**
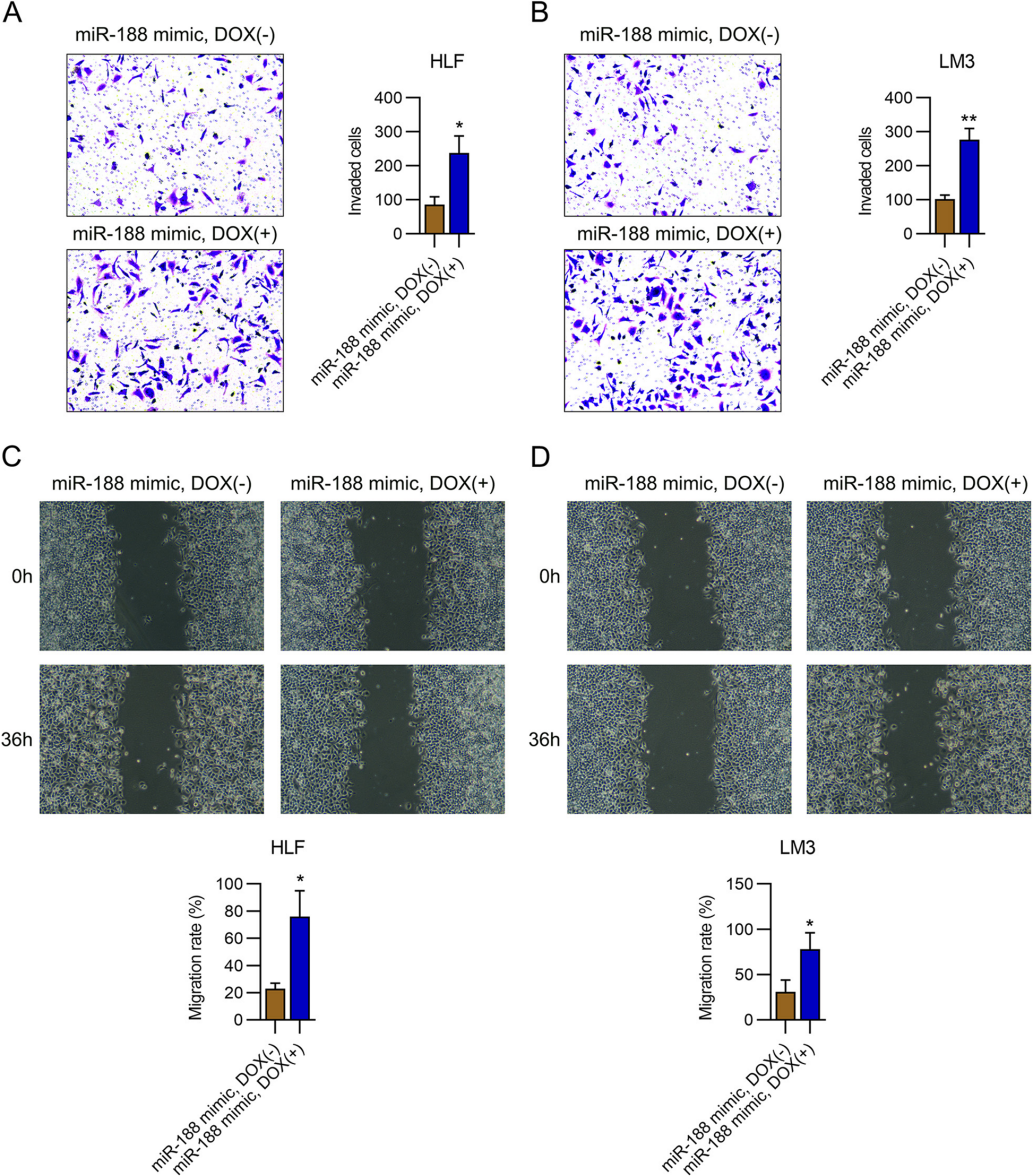
FOXN2 silencing represses the migration of HLF and LM3 cells. HLF and LM3 cells with miR-188 upregulation were treated with doxycycline (Dox, 50 ng/ml). **AB** Transwell invasive assays of HCC cells. **CD** The migratory percentage of cells was determined by wound healing assay. * *P* < 0.05, ** *P* < 0.01

To characterize the function of FOXN2 in cell migration and invasion, we performed a transwell migration and wound healing assay. Treatment with doxycycline resulted in noticeable restoration of the number of invaded cells and migratory rate of HLF and LM3 cells with miR-188 mimic transfection (Fig. 7A, B). These data suggest that miR-188 influences HLF and LM3 cell migration via FOXN2.

### Upregulation of miR-188 inhibits in vivo tumor growth of LM3 cells in nude mice

**Fig. 8:**
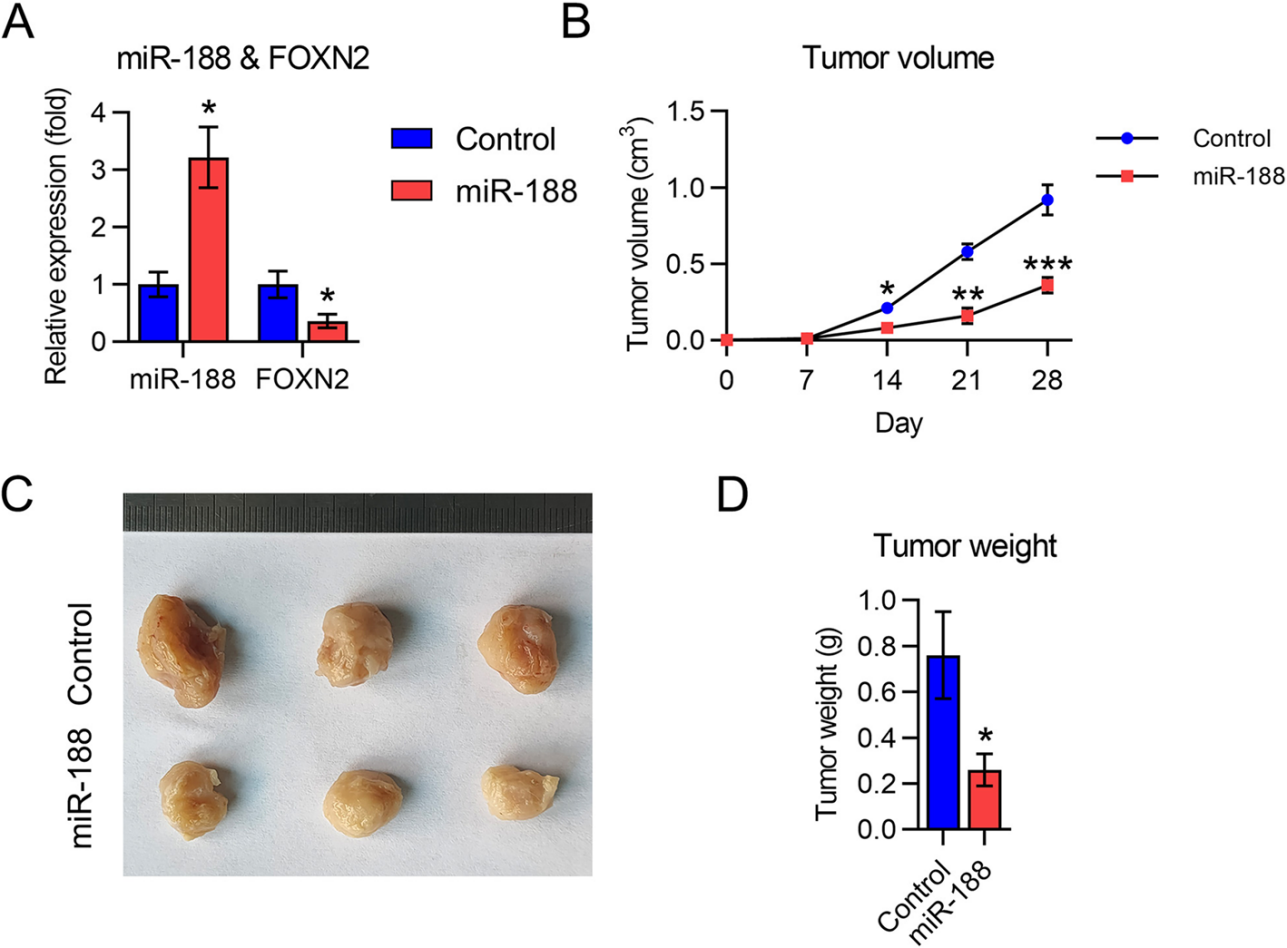
Effect of miR-188 on xenograft hepatocellular carcinoma (HCC) tumorigenesis. LM3 cells stably expressing miR-188 or the control were subcutaneously administered to mice (*n* = 6 in each group). On day 28 post-inoculation, the animals were sacrificed, and tumors were weighed. **A** miR-188 and *FOXN2* expression levels were measured in each group using qPCR. **B** Tumor growth curve at 27 days post-inoculation. **C** Tumor weights on day 28 post-inoculation. **D** Images of tumors from nude mice 28 days post-inoculation. * *P* < 0.05, ** *P* < 0.01, *** *P* < 0.001

Finally, mice were subcutaneously injected with LM3 and LM3-LV-miR-188 cells to examine the role of miR-188 in xenograft HCC tumor growth. miR-188 and *FOXN2* mRNA expression levels in mouse tumor samples (*n* = 6) were also determined. MiR-188 was found to be upregulated, whereas *FOXN2* mRNA expression was reduced in LM3-LV-miR-188-inoculated mice relative to levels in mice in the LM3 infection group (Fig. 8A). The mice were then sacrificed after 28 d, and the tumors were resected and weighed. The results showed that tumors in the LM3-LV-miR-188 group grew more slowly, which was reflected by a lower average tumor volume and weight relative to those in the control group (Fig. 8B-D).

## Discussion

MiRNAs are small regulatory RNA molecules that play a role in the occurrence and development of human cancer and might serve as new treatment targets for cancers [14]. This study showed that low miR-188 expression levels are found in HCC samples and (low) metastatic HCC cell lines, which is closely related to the unfavorable survival of HCC patients. miR-188 overexpression might suppress HCC by downregulating cell proliferation, migration, and invasion via FOXN2. In addition, overexpressed FOXN2 expression was confirmed to have an oncogenic effect on HCC cell growth and migration, counteracting the influence of miR-188 overexpression on HCC cells. These observations showed that miR-188 might serve as an oncogene in HCC occurrence and progression, which needs further investigation for molecular-targeted treatment.miR-188 ectopic expression exists in many types of malignancies. For example, miR-188 levels are decreased in normal acute myeloid leukemia (AML). High miR-188 levels are related to elevated overall survival and event-free survival in patients with normal AML [15]. Low miR-188 levels are also found in metastatic prostate cancer (PC), which could also serve as an independent prognostic factor for unfavorable overall and biochemical non-recurrence survival in patients with PC [16]. Fang et al. indicated that miR-188 expression in HCC samples is decreased and that low miR‑188 expression is closely related to overall and disease-free survival, multiple nodules, and microvascular invasion [9]. The findings of the present study are in agreement with Fang’s report. One of major differences between these two studies and novelty of this study is that we found that FOXN2 also participated in the regulatory role of miR-188 only in metastatic cell lines. Another is that the usage of cell lines in these two studies. Our study majorly focused on the comparison of influence of miR-188 between non-invasive cell lines and metastatic cell lines. miR-188 expression in HCC specimens and cell lines, or more specifically, in metastatic cell lines, was reduced. In our study, the expression of miR-188 in non-invasive cell lines Hep3B and HepG2 was obviously higher than that in (Low-) metastatic cell lines HLF and HCC-LM3. Therefore, the influence of miR-188 on the proliferation, migration, and invasion of both non-invasive and metastatic cell lines was investigated and compared. It was found that miR-188 only exerted its effects on the proliferation, migration, and invasion in metastatic cell lines, such as LM3 and HLF cells, suggesting a differential function of miR-188 in the malignant properties of cancer cells. Moreover, an in vivo tumorigenesis experiment showed that miR-188 upregulation slowed the HCC growth rate in nude mice. These findings confirmed that miR-188 functions as an HCC inhibitor and could be used as a diagnostic and prognostic biomarker for HCC.

The FOX family has different roles in many tumors [17]. FOXN is a subclass of the FOX family and is composed of six members, including FOXN1–6. FOXN2 (forkhead box N2) exists in the nucleus, is encoded at 2p16.3, and is approximately 1296 bp in length. FOXN2 is widely expressed in many organs and tissues in humans [18] and has a critical role in various malignancies, such as breast cancer [19], lung cancer [20], cervical cancer [21], and HCC [22]. For example, low FOXN2 expression predicts poor prognosis in breast cancer. FOXN2 knockdown dramatically promotes breast cancer cell proliferation, migration, and invasion, and represses epithelial–mesenchymal transition [19]. Gain- and loss-of-function experiments have been conducted by Ma et al., and these results indicated that FOXN2 could inhibit cell proliferation and elevate lung cancer radiosensitivity. Moreover, FOXN2 degradation mediated by β-Trcp and RSK2 can facilitate lung cancer occurrence and radioresistance [20]. Liu et al. showed that FOXN2 expression is significantly reduced in both HCC samples and cells. Elevated FOXN2 expression also remarkably suppresses HCC cell proliferation and invasion [22]. These reports indicate a tumor-suppressive role for FOXN2 in cancers. In contrast to previous studies, the present study used doxycycline-inducible FOXN2 silencing to probe the influence of FOXN2 knockdown on HCC cell proliferation and migration (LM3 and HLF). One of differences between Liu’s report and ours was the different cell lines; therefore, it was hypothesized that the effect of FOXN2 is cell lineage-specific. Most importantly, our study identified that miR-188 as an upstream modulator of FOXN2 in regulation of HCC cell properties. Our data showed the expression of FOXN2 is conversely correlated to the level of miR-188 in LM3 and HLF cells. FOXN2 upregulation restored the proliferative and migratory abilities of the LM3 and HLF cells with transfection of miR-188 mimic, suggesting that FOXN2 is essential for the proliferation and migration capacities of LM2 and HLF cells.

## Conclusions

In summary, the results indicated that miR-188 expression in HCC is dramatically decreased. MiR-188 is capable of potently suppressing HCC cell proliferation and migration in vitro and suppressing in vivo tumorigenesis. The tumor-inhibitory role of miR-188 is probably mediated by the targeting of FOXN2. These observations provide new insights into the molecular mechanism of HCC and indicate that miR-188 could function as a promising prognostic biomarker and HCC treatment target.

## Supplementary Information

**Additional file 1.** Raw WB data 5D Figure legend. Raw picture of WB. The raw picture of WB in Fig. 5D are displaying.

**Additional file 2.** Raw WB data 6A Figure legend. Raw picture of WB. The raw picture of WB in Fig. 6A are displaying. 

## Data Availability

The data that support the findings of this study are available from the corresponding author Ya-jie Shao, upon reasonable request.

## Abbreviations

miR-188: MicroRNA-188-5p
HCC: Hepatocellular carcinoma
FOXN2: Forkhead box N2
CFA: Colony formation assay
DLRA: Dual-luciferase reporter assay
AML: Acute myeloid leukemia
PC: Prostate cancer
