# Supplementary figures and images for: MicroRNA-188-5p inhibits hepatocellular carcinoma proliferation and migration by targeting forkhead box N2
Source: BMC Cancer. 2023 Jun 5;23:511. doi: 10.1186/s12885-023-10901-7 (PMC10243064; doi:10.1186/s12885-023-10901-7)

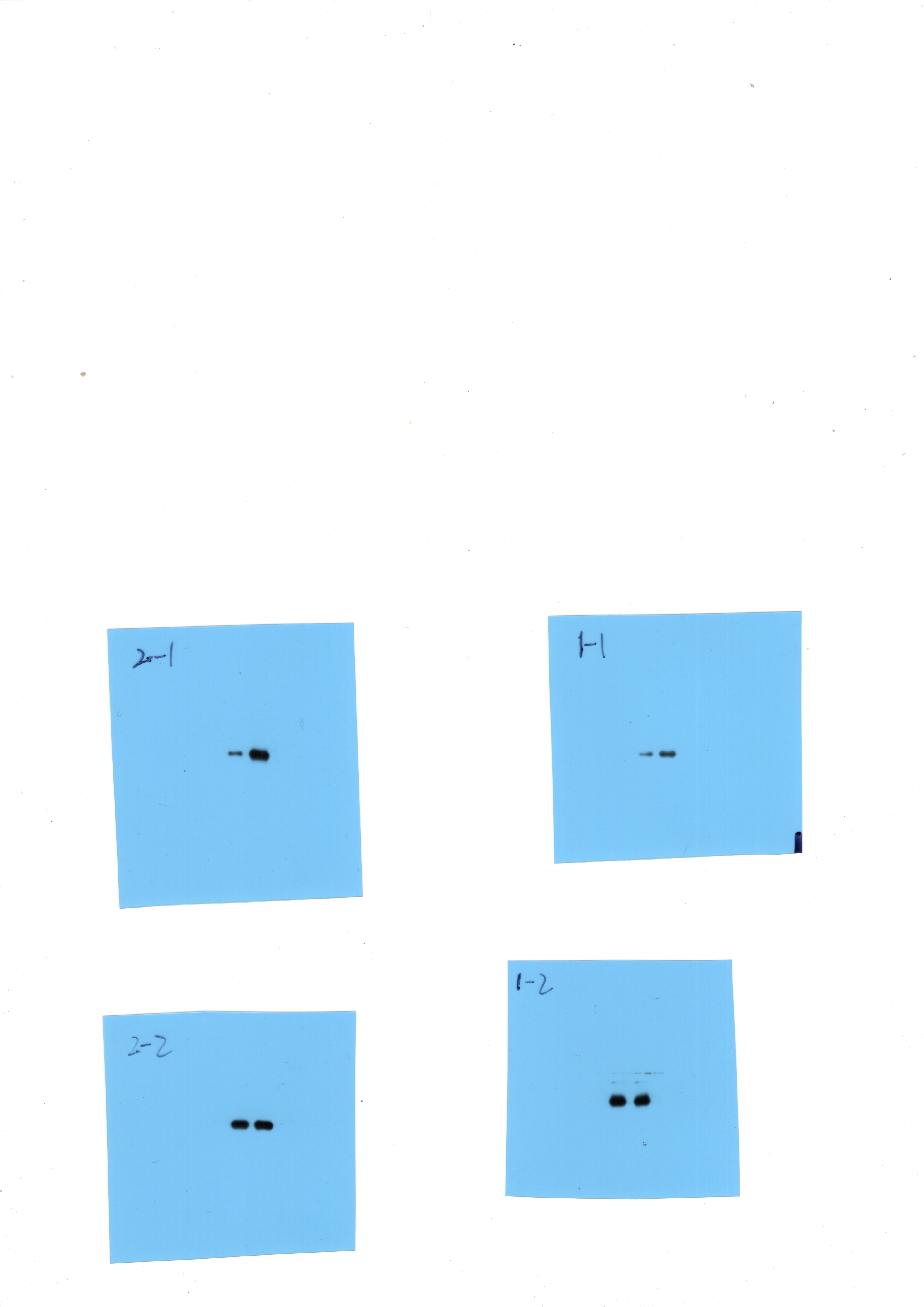

Supplement: Supplementary file 2 — Additional file 2. Raw WB data 6A Figure legend. Raw picture of WB. The raw picture of WB in Fig. 6A are displaying. [file 12885_2023_10901_MOESM2_ESM.tif]
